# ‘Levelling up’ in the UK must involve a reduction in inequalities in children's life chances

**DOI:** 10.1016/j.puhip.2022.100246

**Published:** 2022-03-17

**Authors:** Michelle Black, David Taylor-Robinson, Andrew CK. Lee, Joanne R. Morling

**Affiliations:** School of Health and Related Research (ScHARR), The University of Sheffield, Sheffield, UK; Institute of Population Health, University of Liverpool, Liverpool, UK; School of Health and Related Research (ScHARR), The University of Sheffield, Sheffield, UK; The University of Nottingham, UK

Efforts to reduce inequalities are being pursued by many countries worldwide, for economic, moral, and social reasons. There is good evidence to suggest that a more equal society leads to better economic prospects, more cohesion and better health [1]. Some countries are more unequal than others, with large differences in wealth and health in relation to where people live. The UK is one of the most regionally unbalanced countries in the developed world [2], with inequalities both within and between regions and a stark north-south divide [3]; for every three jobs created in the south of the country there is one job created in the north, furthermore educational assessments at all ages illustrate attainment gaps between the north and the south [4].

The UK government has launched a plan to ‘level up’ [5] the country to redress geographical differences in people's lives, for example in life expectancy, pay, unemployment and transport. Levelling up policy objectives are underpinned by ‘missions’ or targets including areas such as education, skills, wellbeing and healthy life expectancy. The roots of health inequality are in early childhood with socio-economically driven inequalities in child development persisting across the life course, negatively impacting people's future health, wellbeing and life chances, and perpetuating health inequalities into adulthood [6]. Therefore, reducing inequality through addressing these target areas requires a life course approach and this needs to start early in life through families, institutions and places to redress childhood disadvantage which limits educational attainment, employment, physical and mental health outcomes in later life [[7], [8], [9]]. However, the only direct focus on children in the plan is in relation to education with a focus on eliminating illiteracy and innumeracy. Specifically, that by 2030 *90% of all primary school children in England will achieve the expected standard in reading, writing and maths, with the percentage of children meeting the expected standard in the worst performing areas improving by a third.* Education, skills, wellbeing and healthy life expectancy targets will be impossible to hit without a clear plan to address child health and wellbeing.

Can hungry children learn as well as those who are well nourished? Can children exposed to adversities such as parental mental health problems learn as well as those who do not experience early adversity? Can children who receive little support with reading and learning in the home achieve as much academically as those who do? Can children with social and emotional behavioural problems develop their cognitive abilities as well as those who don't? The answer to all of these questions is largely, no [10]. Without a central focus on improving child development, health and reducing child poverty as part of its policy programme, levelling up will be difficult to achieve, given the massive and growing regional inequalities in child wellbeing within the UK.

Regional inequalities in the health and wellbeing of children living in the north of England were laid bare in a recent report, Child of the North [11]. The report shows that children in the North of England are more likely to live in poverty than those in the rest of England (27% compared to 20%) – and increasingly so since the pandemic. Poverty is the lead driver of inequalities between children in the North and their counterparts in the rest of the country, leading to worse physical and mental health outcomes, educational attainment, and lower lifelong economic productivity. To reduce the number of children in poverty the assumption from the UK levelling up plan is that efforts to boost income, employment and places will benefit children indirectly through their parents. But the Child of the North report makes it clear that the starting point for levelling up is to reverse the changes to the welfare system that have led to rising child poverty.

Prioritising child wellbeing, by tackling poverty and family adversity [12], in tandem with a large scale system wide ‘levelling up’ plan could see the transformation that's needed delivered. To be effective a child wellbeing plan would need the necessary legislative powers to enable delivery, adequate funds as well as devolution of those funds, and a workforce working across the system. With regard to legislation, other countries have adopted a child-centred approach. For example, New Zealand has placed the ‘wellbeing’ of children and young people as a central pillar of its economic strategy through its Child Poverty Reduction Act [13]. In the UK, countrywide devolution has enabled different policy approaches to child development [14]. The Welsh government legislates duties related to children's play on local authorities, through the Wellbeing of Future Generations Act 2015. In Scotland a child-centred approach is enshrined in the Getting It Right for Every Child approach and national planning framework, as legislated in the Children and Young People Act 2014. The focus adopted by these countries recognises that transforming children's lives via educational opportunities and poverty reduction is key to reducing socio-economic inequalities in health and the intergenerational transmission of inequality.

The provision of adequate funding and devolution of the decision-making around how those resources are deployed locally could enable the delivery of locally generated child wellbeing programmes that could target issues such as food security, obesity, learning and education, development of capability and skills, and health and wellbeing. This could enable the flow of funds and greater investment in services such as early years providers, schools and mental health services for children. The delivery of any programmes are reliant on a workforce that has the capacity, skills and cross-sector mobility to deliver it. Consequently, national and local action are needed on workforce issues to redress, for example, falling numbers of health visitors [15] and low wages of early years workers [16]. This will need longer term workforce planning in view of the significant lag time in training and developing this workforce.

A laser-like focus on childhood, in tandem with macro-level changes such as those outlined in ‘levelling up’, could lever the change required to transform children's lives by enabling the development of capabilities for better health and wellbeing throughout the life course and redressing intergenerational inequality. We suggest there's a real missed opportunity in the ‘levelling up’ strategy around addressing inequalities in the main drivers of child health. Levelling up through places and people, focusing on the youngest, could be a powerful recipe for a more equal society.
